# Development and validation of a score to identify in the Emergency Department patients who may benefit from a time-critical intervention: a cohort study

**DOI:** 10.1186/s13049-015-0150-y

**Published:** 2015-09-17

**Authors:** Kirsty Challen, Mike Bradburn, Steve W. Goodacre

**Affiliations:** University of Sheffield, Regent Court, Regent Street, Sheffield, S1 4DA UK

## Abstract

**Background:**

Risk stratification methods developed on the basis of predicting illness severity are often used to prioritise patients on the basis of urgency. Illness severity and urgency may not be interchangeable. Severe illness places patients at risk of adverse outcome, but treatment is only urgent if adverse outcome can be prevented by time-sensitive treatment. We aimed to develop a score to identify patients in need of urgent treatment, on the basis of potential to benefit from time-sensitive intervention, and to compare this with a severity score identifying patients at high risk of death.

**Methods:**

A sequential cohort of adults presenting to one Emergency Department by ambulance and admitted to hospital was prospectively collected (2437 derivation, 2322 validation). Data on outcomes representing potential to benefit was collected retrospectively on a random subset (398 derivation, 227 validation). Logistic regression identified variables predictive of death and potential to benefit from urgent treatment.

**Results:**

Death was predicted using age, respiratory rate, diastolic blood pressure, oxygen saturations, temperature, GCS and respiratory disease (AUROC 0.84 (95 % CI 0.8–0.89) derivation and 0.74 (0.69–0.81) validation), while potential to benefit was predicted by pulse, systolic blood pressure and GCS (AUROC 0.74 (0.67–0.80) derivation and 0.71 (0.59–0.82) validation).

**Conclusions:**

A score developed to predict the need for urgent treatment has a different composition to a score developed to predict illness severity, suggesting that triage methods based on predicting severity could lead to inappropriate prioritisation on the intended basis of urgency.

**Electronic supplementary material:**

The online version of this article (doi:10.1186/s13049-015-0150-y) contains supplementary material, which is available to authorized users.

## Introduction

### Background

There were 5.3 million emergency hospital admissions in England in 2012–3, mainly from the 21 % of Emergency Department (ED) attendances resulting in hospital admission [1]. Similarly, in 2010–11, only 71,801 (45 %) of 160,460 critical care admissions were planned [2]. EDs and admissions units therefore need rapid and accurate methods to identify those in urgent need of treatment.

### Importance

Within emergency care illness severity and urgency are often assumed to be interchangeable concepts. However, severity reflects the risk of a poor outcome or impact of a condition upon the patient [3], whereas urgency reflects the potential to benefit from timely care; so a patient with metastatic cancer would be severely ill but a patient with facial angioedema would be of high urgency. A measure of patient urgency should identify patients whose outcome will be improved with prompt care and/or those whose outcome will worsen without this care. We have demonstrated that many risk scores have been developed to predict adverse outcomes not amenable to intervention, and are therefore severity rather than urgency scores [4].

Recent commentary has drawn attention to the disconnect between the identification of patients at risk of a particular outcome and the potential of those patients to benefit from available interventions [5]. Although systems to identify deteriorating patients and responding rapidly are intuitively appealing [6], meta-analysis of these amongst in-patients has failed to identify a benefit in terms of patient outcome [7, 8]. This may reflect use of scores that measure severity rather than urgency.

### Goals

We therefore aimed to develop a score to identify in the ED patients of high urgency with the potential to benefit from time-sensitive interventions, and to compare this with a score to identify patients at high risk of death.

## Methods

### Study design and setting

This study was developed alongside the DAVROS project which developed and validated a risk-adjustment method for research and audit in emergency care and has been described in full elsewhere [9]. Data were collected from February-May (derivation) and October-December (validation) 2008 at Northern General Hospital, Sheffield, the only adult ED serving the half million population of Sheffield. It is an acute teaching hospital with 1100 beds. The ED at the time of the study had 98,000 attendances per year. Interventional cardiology, critical care and acute theatres are all available on site.

### Subjects

Patients were included if they were transported by an emergency ambulance, then either died in the ambulance or ED or were admitted to hospital. Patients who had no vital signs at the time of ambulance arrival (even if resuscitation was attempted) were excluded. Patients aged under 65 with trauma were excluded prospectively as risk prediction in the trauma population has been extensively studied and is strongly influenced by variables such as injury site and type which are not replicated in non-trauma patients (see https://www.tarn.ac.uk). Patients aged over 65 were excluded retrospectively if their reason for admission was purely for trauma. The cohort was restricted to patients presenting by ambulance to ensure a clear point of entry to the emergency care system. Children and patients with purely obstetric presentations were excluded as their physiology is different and therefore predictors of adverse outcome are likely to be different, as are mortality and critical illness rates.

The whole dataset was used to develop the tool to predict death. Patient casenotes were screened in date order until adequate numbers of patients with outcomes of interest were included for the tool to predict potential to benefit. This group constituted the “potential to benefit” subset of the study population.

### Ethical approval

Ethical approval for both studies was obtained from Leeds (East) REC (09/H1306/2). Approval to use patient identifiable data without specific consent was gained from the Patient Information Advisory group for the original study and from the National Information Governance Board (ECC 5-07(f)/2009) for this study.

### Data extraction

Predictor variables were extracted from ED records within 2 days of presentation and entered directly into an online database. Data entry staff were clerical personnel who were specifically trained for the study but had no involvement in the analysis phase. Study staff randomly sampled and rechecked entered data to ensure data quality. Age, blood pressure, Glasgow coma scale, oxygen saturation (breathing air and/or breathing supplemental oxygen), pulse rate, respiratory rate and temperature as recorded on arrival at the ED were chosen as they are widely recorded in a relatively standardised manner. Pulse pressure was calculated from systolic and diastolic blood pressures immediately prior to data analysis. Also extracted from the ED notes was the presence of specific co-morbidities (active malignancy, chronic respiratory disease, heart disease, asthma, diabetes, epilepsy, and warfarin or steroid use), as recorded by the attending clinician. Presenting complaint was recorded verbatim as described to reception staff but not further analysed as there was no way of confirming consistency of recording.

### Outcomes

The patient group of interest in this study is those where urgent intervention had the potential to affect survival, ie where a patient death was prevented or could potentially have been prevented by urgent intervention. We therefore classified 7-day outcomes as:

- inevitable death: the patient died and a decision was made within 24 h of admission not to attempt CPR;
- potentially preventable death, where the patient died but no decision was made to withdraw or limit care;
- potentially prevented death, where the patient survived and received a potentially life-saving intervention (defined below);
- non-critical illness.

The 7 day timescale was selected to achieve a balance between allowing sufficient time for an illness course to declare itself and minimising confounding by chronic disease and/or iatrogenic factors. Mortality was recorded by study staff using hospital record review, supplemented by a search of Coroner’s Office records. It was assumed that all patients admitted to intensive care received potentially life-saving intervention(s) [10, 11]. Interventions that had the potential to prevent death were identified from hospital casenotes by KC using *a priori* definitions (Table 1) based on evidence-based guidelines for acute care (Appendix). No judgement was made as to whether the intervention was helpful in individual cases, and outcome data was abstracted before examination of predictor variable data, to minimise abstractor bias [12]. It must be noted that many of these interventions are not supported by high quality evidence. They are, however, similar to the list of life-saving interventions developed independently and concurrently by another research group [13]. It was not possible to examine primary care records to see if patients were admitted within the 7 days to a different hospital; however given local geography and facilities this was felt to be unlikely.

**Table 1:** Interventions defined a priori as potentially life-saving

Airway interventions
• Use of airway adjunct or procedure to maintain patent airway.
• Use of intravenous/intramuscular adrenaline to treat or prevent airway compromise.
Breathing interventions
• Bag-valve-mask ventilation (unless during procedural sedation), intermittent positive pressure ventilation, or non-invasive ventilation.
• Decompression of tension pneumothorax.
• Drainage of significant pleural effusion (>1 litre).
• Insertion of chest drain for pneumothorax in patients with pre-existing lung disease.
• Intravenous therapy except steroids for asthma.
Circulation interventions
• Cardioversion (chemical or DC) of ventricular tachycardia or supraventricular tachycardia or atrial fibrillation with accessory pathway.
• CPR.
• Emergency endoscopy or surgery for upper GI bleed or use of Sengstaken tube or use of vasopressin/terlipressin.
• Infusion of >2 litres of fluid or transfusion for haemodynamic instability.
• Laparotomy for GI bleed/gynaecological bleed (including ectopic)/AAA.
• Sepsis care bundle.
• Thrombolysis for AMI or PE, or percutaneous revascularisation.
• Therapeutic (not diagnostic) pericardiocentesis.
• Transcutaneous or external pacing or administration of atropine (except in theatre).
• Vasopressor use (except bolus dosing in theatre).
Disability interventions
• Administration of naloxone or flumazenil (unless related to procedural sedation).
• Administration of 10 %/50 % dextrose.
• Administration of >1 dose benzodiazepines/other anticonvulsants for fitting.
• Neurosurgical intervention.
Other interventions
• Active rewarming (not including Bearhugger).
• Laparotomy for sepsis/infarction/obstruction.
• New initiation of renal replacement therapy.
• Specific poisons antidotes including N-acetylcysteine.

### Data analysis

Sample size was based on ten observed outcome events per predictor variable included in the analysis [14]. Given the different frequencies of death and potential to benefit the sample sizes required for the two outcomes therefore varied.

Risk of each outcome in various groupings of predictor variables was explored visually using histograms (data available). Groups of similar risk were collapsed for further analysis. Given the absence of linear and monotonic relationships with risk, we wished to avoid arbitrary dichotomisation of data and therefore avoided decision tree analysis. Univariate association between potential predictor variables and outcome was assessed using logistic regression in SPSS, and first order interaction of variables significant at p < 0.15 was examined. Variables found to be significantly predictive of outcome at p < 0.1 were block entered into the multivariate analysis. Linear coefficients were then recalculated for independently predictive variables and an equation to predict poor outcome generated from those coefficients using the general formula p(outcome) = e^xb^/1 + e^xb^, where xb is the linear predictor.

The equations generated were applied to the validation sets without re-estimation of coefficients and performance assessed using ROC curves.

## Results

### Study subjects

**Fig. 1:**
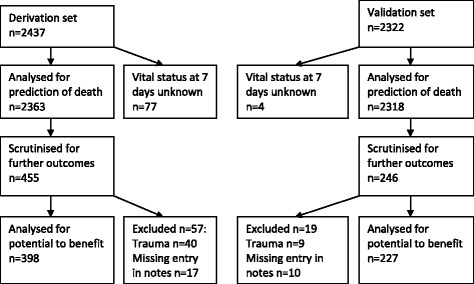
Consort diagram

The derivation cohort included 2437 patients (1131 male, mean age 69) with 398 in the potential to benefit subset (183 male, mean age 66.5), while the validation cohort included 2322 patients (1093 male, mean age 70) with 227 in the potential to benefit subset (95 male, mean age 71.3) (fig 1 for CONSORT diagram). Table 2 compares demographic characteristics of both populations and subsets.

**Table 2:** Patient demographics

	Derivation cohort		Validation cohort	
	Full cohort for death (*n* = 2437)	Subset for potential to benefit (*n* = 398)	Full cohort for death (*n* = 2322)	Subset for potential to benefit (*n* = 227)
Variable	Mean (sd; range)			
Age	69 (19; 18–103)	66.5 (20.2; 18–102)	70 (19; 18–103)	71.3 (18.3; 19–96)
Respiratory rate	19 (6;6–60)	20 (7; 6–45)	20 (6.6; 8–80)	20 (5.8; 12–40)
Systolic BP	136 (29; 24–266)	133 (29; 45–243)	139 (28.4; 44–261)	138 (28.6; 60–249)
Diastolic BP	75 (15; 30–153)	75 (15; 36–130)	76 (15; 11–151)	74 (15; 36–142)
Pulse rate	88 (24; 21–215)	92 (24; 35–188)	88 (23; 20–180)	89 (21.9; 35–152)
Temperature	36.6 (1.2; 26.0–41.0)	36.5 (1.2; 26.0–40.0)	36.5 (1.1; 25.2–40.5)	36.6 (1.40; 26.4–39.6)
Variable	Median (IQR; range)			
SaO2 breathing air	97 (95–98; 50–100)	97 (94–98; 66–100)	97 (95–98; 45–100)	96 (94–98; 45–100)
SaO2 breathing oxygen	98 (95–100; 24–100)	97 (96–99; 71–100)	98 (95–100; 60–100)	98 (96–100; 84–100)
GCS	15 (15–15; 3–15)	15 (15–15; 3–15)	15 (15–15; 3–15)	15 (15–15; 7–15)
Variable	Number (percentage)			
Male	1131 (46.4)	183 (46)	1093 (46.5)	95 (42)
Active malignancy	110 (4.5)	23 (5.8)	96 (4.1)	6 (2.7)
Outcomes	Number (percentage)			
All deaths	128 (5.2)		141 (6.0)	
Inevitable death		15 (3.7)		2 (0.8)
Potentially preventable death		5 (1.2)		1 (0.4)
Prevented death		79 (19.8)		35 (15.4)

### Statistical analysis

After grouping variables and collapsing groups with similar risk, variables significantly predictive of potential to benefit on univariate analysis are in Table 3. Full details of all variables assessed are in Additional file 1: Table S1.

**Table 3:** Univariate logistic regression analysis of predictor variables for potential to benefit

Variable	exp(B)	*P*	95 % CI for exp(B)	
Pulse (ref <71)		<0.001		
71–110	0.522		0.316	0.862
>110	3.756		1.930	7.309
Respiratory rate (ref <16)		<0.001		
16–18	1.009		0.605	1.683
19–23	3.463		1.819	6.594
>23	3.991		2.140	7.443
Systolic BP (ref 121–180)		<0.001		
<100	4.461		2.397	8.302
100–120	2.140		1.250	3.665
>180	31.475		13.479	73.496
Pulse pressure (ref 51–76)		<0.001		
<51	1.791		1.143	2.805
>76	3.540		2.030	6.174
GCS (ref 13–15)		<0.001		
3–8	34.446		16.607	71.447
9–12	2.203		1.235	3.930
SaO2 (ref low risk (99–100 breathing air))		<0.001		
High (<95 breathing air or <96 with supplemental O2)	1.583	0.156	0.839	2.987
Moderate (95–98 breathing air or >95 with supplemental O2)	0.470	0.004	0.283	0.781

Analysis of interactions showed significant interactions in: pulse by respiratory rate, systolic blood pressure and SaO2, respiratory rate by pulse pressure and SaO2, systolic blood pressure by pulse pressure and SaO2 and pulse pressure by SaO2. Briefly it appears that hypoxia confers increased risk if tachycardia is absent or if tachypnoea is present, and that normal pulse pressure confers increased risk in the presence of systolic hypotension.

Multivariate logistic regression analysis including interactions of oxygen saturations with pulse and respiratory rate produced estimated odds ratios (Exp(B)) and *p* values as in Table 4, with pulse, systolic blood pressure and GCS remaining significant. These were block re-entered into a multivariate analysis to develop linear coefficients for the final model:

**Table 4:** Multivariate analysis of predictor variables for potential to benefit

Variable	exp(B)	*p*	95 % CI for exp(B)	
Pulse (ref <71)		0.02		
71–110	0.371		0.034	4.101
>110	5.176		1.125	23.816
Respiratory rate (ref <16)		0.228		
16–18	4.814		0.636	36.443
19–23	19.144		1.035	354.172
>23	6.664		0.723	61.405
Systolic BP (ref 121–180)		0.061		
<100	3.687		1.089	12.481
100–120	1.112		0.407	3.043
>180	2.468		0.615	9.905
Pulse pressure (ref 51–76)		0.536		
<51	1.479		0.548	3.992
>76	1.729		0.611	4.894
GCS (ref 13–15)		<0.001		
3–8	23.013		3.806	139.163
9–12	4.060		1.114	14.790
SaO2 (ref low risk)		0.971		
High	0.946		0.146	6.123
Moderate	0.840		0.164	4.300
Pulse/SaO2 interaction		0.210		
Respiratory rate/SaO2 interaction		0.210		

$$ \mathrm{P}\left(\mathrm{potential}\kern0.5em \mathrm{t}\mathrm{o}\kern0.5em \mathrm{benefit}\right)={\mathrm{e}}^{\mathrm{xb}}/1+{\mathrm{e}}^{\mathrm{xb}} $$

where xb = (pulse[71–110]*2.373) + (pulse[>110]*9.533) + (sbp[<100]*3.614) + (sbp[100-120]*1.488) + (sbp[>180]*2.888) + (gcs[3-8]*13.28) + (gcs[9-12]*2.770).

The performance of this equation was assessed using a ROC curve, which had an area under the curve (c-statistic) of 0.737 (95 % CI 0.671–0.804). When applied to the validation set it had an area under the curve (c-statistic) of 0.707 (95 % CI 0.594–0.820).

The same process when performed to predict all deaths at 7 days (multivariate analysis in Additional file 1: Table S2, full details available) generated the equation below. Of note is the inclusion of age, temperature and co-morbidity which predict death but not potential to benefit.

$$ \mathrm{P}\left(\mathrm{death}\right)={\mathrm{e}}^{\mathrm{xb}}/1+{\mathrm{e}}^{\mathrm{xb}} $$

where xb = (age[50–69]*0.608) + (age[70–85]*0.348) + (age[>85]*0.924) + (respiratory rate[19-23]*1.753) + (respiratory rate[>23]*2.791) + (DBP[<65]*3.172) + (DBP[>90]*5.204) + (sao2[high risk]*3.202) + (sao2[moderate risk]*0.932) + (temperature[<36]*4.714) + (GCS[3-12]*29.372) + (GCS[13-14]*5.311) + (respiratory disease [present]*12.355) + (respiratory disease [present] and temperature[<36]*7.466).

This had a c-statistic of 0.847 (95 % CI 0.8–0.894). When applied to the validation set, the equation had an area under the curve (c-statistic) of 0.741 (95 % CI 0.685–0.806).

## Discussion

We have demonstrated that a combination of pulse, systolic blood pressure and Glasgow Coma Score can predict provision of time-sensitive interventions with moderate discrimination. Different variables (age, respiratory rate, diastolic blood pressure, oxygen saturations, temperature, Glasgow Coma Score and a pre-existing diagnosis of respiratory disease) predict death within seven days. Thus a score developed to predict the need for urgent treatment will differ from a score developed to predict illness severity, suggesting that triage methods based on severity could lead to inappropriate prioritisation on the intended basis of urgency.

### Strengths

This is the first study specifically to address the identification of at-risk patients in an ED population not preselected for diagnosis or severity, and to use the provision of a life-saving intervention as an outcome measure. It therefore addresses the issues facing emergency clinicians more closely than the existing literature.

Although our definitions of time-sensitive interventions might be criticized for being defined arbitrarily, the concept of an “Emergency Care Sensitive Condition” is being more widely embraced and supports our methodology [15]. It is apparent that patterns of physiological derangement differ between patients who die and those who receive life-saving intervention [16]. The development of a tool to identify this second group was therefore necessary.

### Limitations

Patients who were not admitted to hospital and those who self-presented were excluded from the initial study dataset as the aim was to develop a risk-adjustment tool for emergency admissions to hospital. This limits the generalisability of this study in terms of developing a clinical score. Ideally a full cohort of presenting patients would be studied and those discharged from the ED followed up to analyse post-discharge adverse events. However that is logistically unfeasible, as rates of short-term death after discharge from the ED are 30–50/100,000 [17, 18], and only 11 % of self-presenting patients are admitted to hospital [1], so the required cohort size would have been impractical. Obviously our findings cannot be applied to other patient groups such as children or trauma patients.

Our definition of an inevitable death might be considered overly restrictive but we chose this deliberately to include the widest possible group as having potential to benefit.

As a single-site study it may be that these results are not generalisable; interpretation of vital sign derangement is not only affected by patient factors but also by the health care system, staffing levels and types and time available for patient care [19]. Thus a process of external validation might find that life-saving interventions are provided differently in other settings.

We initially wished to examine the role of clinician gestalt in detection of the at-risk patient. Early data collection included a “yes/no” question to the transporting paramedic as to whether the patient was critically ill; this had to be abandoned due to poor rates of completion.

### Implications for clinical practice and policy

The variables we have identified as predictive of a need for life-saving intervention are not the same as those in use in many standardized early warning scores. This may reflect the inappropriateness of developing early warning scores using data sets in which death is the main outcome. This score may be more appropriate than existing scores but should not yet be widely applied in standard practice. It needs wider validation, ideally including comparison with unstructured clinician (doctor or triage nurse) gestalt and with NEWS as the currently mandated standard of care. There must also be consideration as the score is applied of whether the outcomes used to develop definitions of urgency are still valid; the interventions listed in the Appendix are acknowledged to be based on incomplete evidence; it is to be hoped that as the evidence base for emergency care is developed these can be refined (for example, intravenous magnesium in acute severe asthma would no longer be considered a potentially life-saving intervention [20]).

These results highlight the potential flaws in applying clinical scores to predict outcomes other than those for which they were originally derived. As the health economist Tony Culyer said “capacity to benefit is not identical to need” [21], and clinicians should be clear about the reasons for which a score is being used. Equally, if a scoring system is used as casemix adjustment in an attempt to assess or improve quality, it should be clear that it identifies conditions which are amenable to alteration with good care [15].

This leaves the working emergency clinician in the situation of not having a score developed for and demonstrated to work in the emergency setting. There are two options: firstly to use an existing score but to recognise its limitations in the ED; secondly not to use a score but to rely on the unstructured judgement of clinical staff. In the current culture of high regard for standardised paperwork easily amenable to retrospective audit this is unlikely to be managerially palatable. Given the current state of equipoise over the utility of standardised scores in terms of patient benefit clinicians should also be encouraged to participate in formal research to address the issue.

### Implications for research

Researchers in other settings have demonstrated the value of changes in physiological scores in prognostication [22]; ideally ongoing research would examine the prognostic value of response (or non-response) to treatment provided prehospitally or in the ED.

We have defined *a priori* a group of interventions which appear on best available evidence to be potentially life-saving and time-sensitive. However, the evidence supporting these is incomplete and our beliefs underlying many frequently-used interventions would bear further scrutiny; patients who could benefit could then be more reliably identified. We made no attempt to assess functional outcome of those patients defined as having potential to benefit; future researchers may wish to consider whether morbidity should also be a component of benefit.

Identification of and response to the patient at risk requires more than a reliable scoring system; complex psychosocial and cognitive factors affect decision-making, particularly in the pressured ED environment [23], and examination of the interaction of these factors should be a priority for future research [24].

In summary, we have developed a score that predicts urgency (potential to benefit from time-sensitive treatment) with moderate discrimination. The score has different constituent variables and weights than a score developed to measure severity (risk of death). Early warning scores developed to predict death may not be useful predictors of urgency.

## Appendix

### Acute care guidelines used to identify potentially lifesaving interventions

- Acute illness: NICE clinical guideline 50
- Acute onset atrial fibrillation: NICE clinical guideline 36, SIGN 94 and 129
- Alcohol-related disease: NICE clinical guideline 100
- Anaphylaxis: College of Emergency Medicine
- Arrhythmia: SIGN 94
- Asthma: SIGN 101
- Cardiac arrest: SIGN 94
- COPD: NICE clinical guideline 101
- DVT: NICE clinical guideline 144 and SIGN 122
- Headache: College of Emergency Medicine and American College of Emergency Physicians
- Hypertension: American College of Emergency Physicians
- LVF: American College of Emergency Physicians and European Society of Cardiology
- NSTEMI/Unstable angina: NICE clinical guideline 94, SIGN 93, American College of Emergency Physicians and European Society of Cardiology
- PE: NICE clinical guideline 144, SIGN 122, American College of Emergency Physicians and European Society of Cardiology
- Pneumonia: American College of Emergency Physicians and British Thoracic Society
- Pneumothorax (spontaneous): British Thoracic Society
- Poisoning: NICE clinical guideline 16, American College of Emergency Physicians and College of Emergency Medicine
- Seizure: American College of Emergency Physicians and College of Emergency Medicine
- Sepsis: NICE clinical guideline 151
- STEMI: SIGN 93 and European Society of Cardiology
- Stroke/TIA: NICE clinical guideline 68, SIGN 108.
- Transient loss of consciousness: NICE clinical guideline 109
- Type 1 diabetes: NICE clinical guideline 15.
- Upper GI bleeding: NICE clinical guideline 141, SIGN 105
- Urinary tract infection: SIGN 88

## Additional file

Additional file 1:
**Development and validation of a score to identify patients who may benefit from a time-critical intervention in the Emergency Department.** (DOCX 88 kb)
