# Supplementary material for: Development and validation of a score to identify in the Emergency Department patients who may benefit from a time-critical intervention: a cohort study
Source: Scand J Trauma Resusc Emerg Med. 2015 Sep 17;23:67. doi: 10.1186/s13049-015-0150-y (PMC4574211; doi:10.1186/s13049-015-0150-y)
Supplement: Additional file 1: — Development and validation of a score to identify patients who may benefit from a time-critical intervention in the Emergency Department. (DOCX 88 kb) [file 13049_2015_150_MOESM1_ESM.docx]

Development and validation of a score to identify patients who may benefit from a time-critical intervention in the Emergency Department

Supplemental tables and figures

Supplemental figure 1: ROC curve for potential to benefit at 7 days (derivation set)

Supplemental figure 2: ROC curve for potential to benefit at 7 days (validation set)


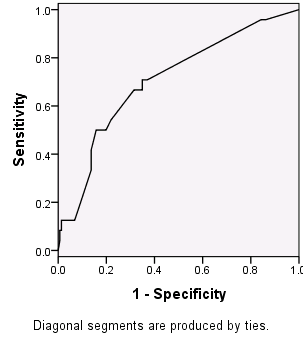


Supplemental figure 3: ROC curve for prediction of death at 7 days (derivation set)


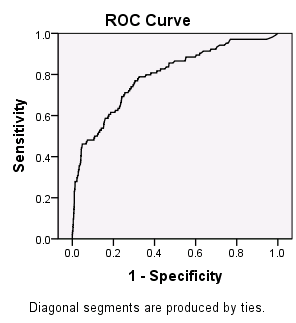


Supplemental figure 4: ROC curve for prediction of death at 7 days (validation set)


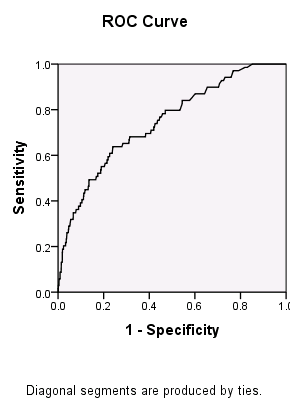


*Table S1: Univariate analysis of variables to predict potential to benefit*

| **Variable** | **Potential to benefit** | | **Total** |
| --- | --- | --- | --- |
| Pulse p<0.001 | | | |
| *<71* | 6 (8) | | 76 |
| *71-82* | 15 (20) | | 76 |
| *83-95* | 16 (20) | | 80 |
| *96-110* | 11 (14) | | 81 |
| *>110* | 34 (44) | | 77 |
| *Missing* | 2 (25) | | 8 |
| Respiratory rate p=0.016 | | | |
| *<16* | 7 (12) | | 59 |
| *16-17* | 17 (21) | | 80 |
| *18* | 13 (17) | | 76 |
| *19-23* | 15 (25) | | 60 |
| *>23* | 25 (35) | | 72 |
| *Missing* | 7 (14) | | 51 |
| Systolic BP p=0.003 | | | |
| *Very low (<100)* | 15 (36) | | 42 |
| *Low (100-120)* | 22 (26) | | 84 |
| *Normal (120-180)* | 37 (15) | | 244 |
| *High (>180)* | 7 (39) | | 18 |
| *Missing* | 3 (30) | | 10 |
| Pulse pressure p=0.005 | | | |
| *<40* | 21 (28) | | 76 |
| *40-50* | 26 (30) | | 86 |
| *51-62* | 7 (10) | | 72 |
| *63-76* | 10 (13) | | 75 |
| *>76* | 16 (21) | | 77 |
| *Missing* | 4 (33) | | 12 |
| GCS p=0.015 | | | |
| *3-5* | 2 (66) | | 3 |
| *6-8* | 4 (57) | | 7 |
| *9-12* | 7 (33) | | 21 |
| *13-14* | 10 (20) | | 49 |
| *15* | 49 (18) | | 275 |
| *Missing* | 12 (28) | | 43 |
| Oxygen saturations breathing air p=0.056 | | | |
| *Very low (<90)* | 10 (32) | | 31 |
| *Low (90-3)* | 8 (33) | | 24 |
| *Normal (94-100)* | 39 (17) | | 231 |
| *Missing* | 27 (24) | | 112 |
| Oxygen saturations breathing supplemental oxygen p=0.439 | | | |
| *<96* | 7 (32) | | 22 |
| *96-99* | 16 (25) | | 63 |
| *100* | 4 (19) | | 21 |
| *Missing* | 57 (20) | | 292 |
| Age population quintiles p=0.039 | | | |
| *<45* | | 22 (28) | 78 |
| *45-64* | | 15 (19) | 79 |
| *65-75* | | 21 (29) | 72 |
| *76-85* | | 18 (19) | 97 |
| *>85* | | 8 (11) | 72 |

| Gender p=0.194 | | |
| --- | --- | --- |
| *Female* | 39 (18.5) | 211 |
| *Male* | 45 (24.6) | 183 |
| *Missing* | 0 | 4 |
| Active malignancy p=0.653 | | |
| *No active malignancy* | 80 (21.3) | 375 |
| *Active malignancy* | 4 (17.4) | 23 |

Table S2: Multivariate analysis of variables to predict all deaths at 7 days

|  | **p** | **Exp(B)** | **95% CI for Exp(B)** | |
| --- | --- | --- | --- | --- |
| Age (ref <50) | <0.001 |  |  |  |
| *50-69* | 0.124 | 0.608 | 0.322 | 1.147 |
| *70-85* | <0.001 | 0.348 | 0.201 | 0.603 |
| *>85* | 0.787 | 0.924 | 0.520 | 1.642 |
| Respiratory rate (ref <19) | <0.001 |  |  |  |
| *19-23* | 0.012 | 1.753 | 1.129 | 2.721 |
| *>23* | <0.001 | 2.791 | 1.730 | 4.5 |
| Diastolic BP (ref 65-90) | <0.001 |  |  |  |
| *<65* | <0.001 | 3.172 | 2.090 | 4.815 |
| *>90* | <0.001 | 5.204 | 3.250 | 8.333 |
| SaO2 (ref low risk >95 breathing air) | <0.001 |  |  |  |
| *High risk (<90 air/<95 O2)* | <0.001 | 3.202 | 1.956 | 5.243 |
| *Moderate risk (90-5 air/ >94 O2)* | 0.754 | 0.932 | 0.598 | 1.452 |
| Temperature <36 | <0.001 | 4.714 | 3.104 | 7.158 |
| GCS (ref 15) | <0.001 |  |  |  |
| *3-12* | <0.001 | 29.372 | 18.674 | 46.197 |
| *13-14* | <0.001 | 5.311 | 3.403 | 8.291 |
| Respiratory disease present | <0.001 | 12.355 | 8.202 | 18.609 |
| Respiratory disease and temperature <36 | <0.001 | 7.466 | 3.134 | 17.784 |
